# Letter Addressed to Baron Liebig, Professor at the University of Giessen

**Published:** 1846-09

**Authors:** Aug. Laurent, Ch. Gerhardt

**Affiliations:** Corresponding Member of the Academy of Sciences; Professor to the Faculty of Sciences, Montpelier


BILLET DOUX TO BARON LIEBIG.

*Letter addressed to Baron Liebig, Professor at the University of Giessen.* By MM. LAURENT and GERHARDT.

*Monsieur le Baron,*—You have just published a brochure, in which you have attacked our labours in the most injurious manner.

You are at liberty, doubtless, to find our labours to be bad, and you may also attack us in a style at which any well bred man would blush; you alone are responsible for your own expressions.

But you have no right to calumniate us; you have no right to designate us forgers, highway robbers, to say, in the face of the world, that in order to refute your theories, to expose your errors, we have deceived the Academy of Sciences, in presenting to that body, on the 22nd of September last, experiments which do not exist, which we never made.

Were you a Frenchman, Monsieur le Baron, we might call you to account for these calumnies, before the legal tribunals, for those experiments were made in Paris, in the laboratory of M. Pelouze, for the most part in the presence of M. Pelouze, or his pupils, and upon substances furnished by that chemist, and also in Montpelier, in the laboratory of the Faculty of Sciences, partly with materials furnished by M. Pelouze, and partly on products prepared in the latter laboratory, and which were shown to many persons, amongst others to the Dean of the Facnlty.

This will serve to show the value of your assertions.

Since your being a foreigner insures you impunity, we have no other resource against you than an appeal to public opinion; we beg you, therefore, Monsieur le Baron, to have the goodness to give our reclamation its right, by retracting your words, and to accept our *salutations empressées.*

AUG. LAURENT,
Corresponding Member of the Academy of Sciences.
CH. GERHARDT.
Professor to the Faculty of Sciences, Montpelier.

*London Lancet.*

